# Supplementary material for: Preparation of Water-in-Oil Nanoemulsions Loaded with Phenolic-Rich Olive Cake Extract Using Response Surface Methodology Approach
Source: Foods. 2022 Jan 20;11(3):279. doi: 10.3390/foods11030279 (PMC8834604; doi:10.3390/foods11030279)
Supplement: Supplementary file 1 [file foods-11-00279-s001.zip › foods-1533448-supplementary.pdf]

Supplementary Material

Preparation of Water-in-Oil Nanoemulsions Loaded with Phenolic-Rich Olive Cake Extract using Response Surface Methodology Approach

S. Mehdi Niknam, Mansoore Kashaninejad, Isabel Escudero, M. Teresa Sanz, Sagrario Beltrán and José M. Benito*

Department of Biotechnology and Food Science (Chemical Engineering Section), University of Burgos, Plaza Misael Bañuelos s/n, 09001 Burgos, Spain; snx1002@alu.ubu.es (S.M.N.); mkx1002@alu.ubu.es (M.K.); iescuder@ubu.es (I.E.); tersanz@ubu.es (M.T.S.); beltran@ubu.es (S.B.)
* Correspondence: jmbmoreno@ubu.es; Tel.: 34-947258810

Supplementray Data S1: Characterization of W/O nanoemulsions

Formulated nanoemulsions were characterized following several methods described in previous works [16,21,23,32,33]. Measurements were performed at least by triplicate.

Droplet size distribution, mean droplet diameter and polydispersity index (PDI) of samples were measured at 25 °C by dynamic light scattering (DLS) using a Zetasizer Nano ZS apparatus (Malvern Instruments Ltd., Malvern, UK).

Total phenolic content (TPC) was measured using Folin-Ciocalteu standard method with some modifications [16] and it was expressed as milligrams of gallic acid equivalents per gram of olive cake (mg GAE/g).

Antioxidant activity (AA) of the samples, expressed as milligrams of Trolox equivalents per liter of sample (mg Trolox/L), was measured by the DPPH free radical scavenging activity method described by Shen et al. [34].

The percentage of phenolic compounds held within the internal aqueous phase of the nanoemulsion after 30 days of storage was measured following the method proposed by Regan and Mulvihill [35]. Thus, 3 g of optimal nanoemulsions were mixed with 3 g of phosphate buffer solution (pH 7) and centrifuged (Eppendorf 5804 centrifuge) at 5,600 × *g* for 90 min. Then, the aqueous phase was collected carefully for TPC and AA analysis, and the percentage of encapsulated compounds (E) was calculated by using Equation (1):

$$\mathrm{E}(\%) = \left(1 - \frac{\mathrm{C}_2}{\mathrm{C}_1}\right) \times 100 \tag{1}$$

where C_2 is the concentration of phenolic compounds found in the outer aqueous phase after centrifugation and C_1 is the initial concentration of phenolic compounds in the inner aqueous phase of the nanoemulsion.

Stability of W/O emulsions was measured in terms of their droplet growth ratio. Since emulsions tend to aggregate during storage, the droplet size of the emulsions was measured after 1 day and also 30 days after preparation. Two different storage conditions were evaluated: 4 °C and room temperature in darkness. In addition, optical characterization of the optimal nanoemulsion was done for 30 days at 25 °C by static multiple light scattering (S-MLS) using a Turbiscan Lab Expert equipment (Formulaction Co., L'Union, France) [23,36,37].

Table S1. Factors (independent variables) and levels for the three experimental designs.

W/O Emulsion Formulation			
Level	Aqueous Phase Content (X_1, % *w*/*w*)	Surfactant Content (X_2, % *w*/*w*)	HLB number (X_3)
1	2	2	3
2	11	11	7
3	20	20	11
**W/O Emulsion Preparation by Rotor-Stator Mixing**			
Level	Rotation Speed (X_1, rpm)	Time (X_2, min)	
1	11,000	5	
2	20,000	10	
3	29,000	15	
**W/O Emulsion Preparation by Ultrasonic Homogenization**			
Level	Time (X_1, min)	Amplitude (X_2, %)	
1	5	20	
2	10	40	
3	15	60	

References (they have the same number that also appears in the main text and in the reference list)

16. Niknam, S.M.; Kashaninejad, M.; Escudero, I.; Sanz, M.T.; Beltrán, S.; Benito, J.M. Valorization of olive mill solid residue through ultrasound-assisted extraction and phenolics recovery by adsorption process. *J. Clean. Prod.* **2021**, *316*, 128340. https://doi.org/10.1016/j.jclepro.2021.128340
21. Gutiérrez, G.; Matos, M.; Benito, J.M.; Coca, J.; Pazos, C. Preparation of HIPEs with controlled droplet size containing lutein. *Colloids Surf. A Physicochem. Eng. Asp.* **2014**, *442*, 111–122. https://doi.org/10.1016/j.colsurfa.2013.05.077
23. Niknam, S.M.; Escudero, I.; Benito, J.M. Formulation and preparation of water-in-oil-in-water emulsions loaded with a phenolic-rich inner aqueous phase by application of high energy emulsification methods. *Foods* **2020**, *9*, 1411. https://doi.org/10.3390/foods9101411
32. Alonso, L.; Roque, L.; Escudero, I.; Benito, J.M.; Sanz, M.T.; Beltrán, S. Solubilization of Span 80 niosomes by sodium dodecyl sulfate. *ACS Sustainable Chem. Eng.* **2016**, *4*, 1862–1869. https://doi.org/10.1021/acssuschemeng.6b00148
33. Roque, L.; Fernández, M.; Benito, J.M.; Escudero, I. Stability and characterization studies of Span 80 niosomes modified with CTAB in the presence of NaCl. *Colloids Surf. A Physicochem. Eng. Asp.* **2020**, *601*, 124999. https://doi.org/10.1016/j.colsurfa.2020.124999
34. Shen, Q.; Zhang, B.; Xu, R.; Wang, Y.; Ding, X.; Li, P. Antioxidant activity in vitro of the selenium-contained protein from the Se-enriched *Bifidobacterium animalis* 01. *Anaerobe* **2010**, *16*, 380–386. https://doi.org/10.1016/j.anaerobe.2010.06.006
35. Regan, J.O.; Mulvihill, D.M. Water soluble inner aqueous phase markers as indicators of the encapsulation properties of water-in-oil-in-water emulsions stabilized with sodium caseinate. *Food Hydrocoll.* **2009**, *23*, 2339–2345, https://doi.org/10.1016/j.foodhyd.2009.06.009
36. Allende, D.; Cambiella, A.; Benito, J.M.; Pazos, C.; Coca, J. Destabilization-enhanced centrifugation of metalworking oil-in-water emulsions: Effect of demulsifying agents. *Chem. Eng. Technol.* **2008**, *31*, 1007–1014. https://doi.org/10.1002/ceat.200700018
37. Rebolleda, S.; Sanz, M.T.; Benito, J.M.; Beltrán, S.; Escudero, I.; González-Sanjosé, M.L. Formulation and characterisation of wheat bran oil-in-water nanoemulsions. *Food Chem.* **2015**, *167*, 16–23. https://doi.org/10.1016/j.foodchem.2014.06.097
